# Non-typhoidal *Salmonella* blood stream infection in Kuwait: Clinical and microbiological characteristics

**DOI:** 10.1371/journal.pntd.0007293

**Published:** 2019-04-15

**Authors:** M. John Albert, Dieter Bulach, Wadha Alfouzan, Hidemasa Izumiya, Glen Carter, Khaled Alobaid, Fatemah Alatar, Abdul Rashid Sheikh, Laurent Poirel

**Affiliations:** 1 Department of Microbiology, Faculty of Medicine, Kuwait University, Jabriya, Kuwait; 2 Microbiological Diagnostic Unit, Public Health Laboratory, Peter Doherty Institute for Infection and Immunity, The University of Melbourne, Victoria, Australia; 3 Microbiology Unit, Department of Laboratories, Al Farwaniya Hospital, Sabah Al-Nasser, Kuwait; 4 National Institute of Infectious Diseases, Tokyo, Japan; 5 Department of Microbiology, Al Amiri Hospital, Sharq, Kuwait; 6 Microbiology Unit, Muabarak Al Kabeer Hospital, Jabriya, Kuwait, and; 7 Department of Medicine, University of Fribourg, Switzerland; Beijing Institute of Microbiology and Epidemiology, CHINA

## Abstract

Non-typhoidal *Salmonella* (NTS) bacteremia is a significant cause of morbidity and mortality worldwide. It is considered to be an emerging and neglected tropical disease in Africa. We studied this in two tertiary hospitals–Al Farwaniya and Al Amiri–in Kuwait, a subtropical country, from April 2013-May 2016. NTS bacteremia was present in 30 of 53,860 (0.75%) and 31 of 290,36 (1.33%) blood cultures in the two hospitals respectively. In Al Farwaniya hospital, one-third of the patients were from some tropical developing countries of Asia. About 66% of all patients (40/61) had diarrhea, and of these, 65% had the corresponding blood serovar isolated from stool culture. A few patients had *Salmonella* cultured from urine. Patients were either young or old. Most of the patients had co-morbidities affecting the immune system. Two patients each died in both hospitals. The number of different serovars cultured in each hospital was 13, and most infections were due to *S*. Enteritidis (all sequence type [ST]) 11) and *S*. Typhimurium (all ST19) except in a subgroup of expatriate patients from tropical developing countries in Al Farwaniya hospital. About a quarter of the isolates were multidrug-resistant. Most patients were treated with a cephalosporin with or without other antibiotics. *S*. Enteritidis and *S*. Typhimurium isolates were typed by pulsed field-gel electrophoresis (PFGE) and a selected number of isolates were whole-genome sequenced. Up to four different clades were present by PFGE in either species. Whole-genome sequenced isolates showed antibiotic-resistance genes that showed phenotypic correlation, and in some cases, phenotypes showed absence of specific genes. Whole-genome sequenced isolates showed presence of genes that contributed to blood-stream infection. Phylogeny by core genome analysis showed a close relationship with *S*. Typhimurium and *S*. Enteritidis from other parts of the world. The uniqueness of our study included the finding of a low prevalence of infection, mortality and multidrug-resistance, a relatively high prevalence of gastrointestinal infection in patients, and the characterization of selected isolates of *S*. Typhimurium and *S*. Enteritidis serovars by whole-genome sequencing that shed light on phylogeny, virulence and resistance. Similarities with studies from developing countries especially Africa included infection in patients with co-morbidities affecting the immune system, predominance of *S*. Typhimurium and *S*. Enteritidis serovars and presence of drug-resistance in isolates.

## Author summary

*Salmonella* organisms are classified into typhoidal *Salmonella* (causing enteric fever) and non-typhoidal *Salmonella* (NTS) (causing infections other than enteric fever). Apart from causing other infections, NTS causes blood-stream infection (bacteremia and septicemia). NTS blood stream infection (NTS-BI) is considered to be an emerging and neglected tropical disease in Africa. It causes a very high morbidity and mortality in Africa. The individuals affected in Africa are children, malnourished people, patients with malaria or HIV etc. These conditions affect the immune system and make them vulnerable to infection with NTS. In these patients, diarrheal disease due to NTS is rare. The majority of infections are due to two types of NTS: Typhimurium and Enteritidis. There is a very high prevalence of multidrug-resistance in NTS making the infection difficult to treat. NTS-BI is also present in other parts of the world including developed countries albeit at a lower prevalence. Kuwait is a high-income, subtropical country in transition (from a developing to developed country), located in the Middle East. We studied NTS-BI in Al Farwaniya and Al Amiri hospitals in Kuwait during April 2013 to May 2016. Out of nearly 30,000 to more than 50,000 blood cultures done in these hospitals, NTS was present in 0. 75 to 1.33% of blood cultures, representing a very small proportion of blood cultures, unlike in Africa. This showed that 31 patients in Al Farwaniya hospital and 30 patients in Al Amari hospital had NTS-BI. Most of these patients had underlying illnesses such as diabetes, lung infection, cancer etc. that affect the immune system, as in Africa. Many patients also had diarrheal disease caused by the same NTS that caused blood stream infection, unlike in Africa. Only two patients in each hospital died, a low mortality, unlike in Africa. The majority of the isolates belonged to Typhimurium and Enteritidis as in Africa. Even though resistance to drugs was a problem, about quarter of the isolates only were multidrug-resistant, a lower prevalence compared to in Africa. In Kuwait, we performed a detailed genetic study of a selected number of Typhimurium and Enteritidis isolates by a modern technique called whole genome sequencing. This revealed genetic determinants encoding drug-resistance and virulence causing blood-stream infection. This type of study was not performed in African isolates. Thus, our study revealed similarities and differences with studies of NTS-BI in Africa.

## Introduction

*Salmonella enterica* subspecies *enterica* serovar Typhi and *Salmonella* Paratyphi A, B and C cause enteric fever, a systemic febrile illness that occurs only in humans. There are more than 2500 serovars of non-typhoidal Salmonella (NTS). NTS infects a variety of hosts and are frequently zoonotic in origin [1]. It mainly causes self-limiting gastroenteritis in humans. NTS has also been recognized as a major cause of extra-intestinal invasive bacterial infection in young children and immunocompromised patients worldwide [2,3]. It is a cause of severe bacteremia [1]. It has been estimated that globally about 3.4 million cases of bacteremia due to NTS occur every year [4]. The estimated worldwide mortality from NTS infection is 155,000 per year [1]. Invasive NTS disease is considered to be an emerging and neglected tropical disease in Africa [1].

Kuwait is a high-income subtropical country situated in the Middle East. It is neither a developing nor a developed country, but is a country in transition. There are no systemic studies of bacteremia due to NTS in Kuwait even though NTS is a major cause of diarrhea in Kuwait [5,6]. Antimicrobial resistance is also a problem among diarrheal stool isolates of NTS in Kuwait [7]. Also, there are no data on serovars of NTS causing infection in Kuwait. Therefore, the primary objective of the study was to assess the case-fraction of NTS bacteremia among bacteremia cases in two tertiary hospitals in Kuwait. The secondary objective was to determine the serovars of NTS isolates and their antibiotic susceptibilities. A study was conducted in two tertiary hospitals in Kuwait. We determined the serovars of NTS isolates and their antibiotic susceptibilities. Moreover, all isolates of *S*. Enteritidis and *S*. Typhimurium serovars were typed by pulsed-field gel electrophoresis (PFGE) to study their relatedness. In addition, selected isolates were subjected to whole genome sequencing for further characterization. We also compared our results with those from Africa. The results are presented in this communication.

## Materials and methods

**Patients**. The only criterion for inclusion in the study was the isolation of a NTS from blood culture irrespective of any accompanying conditions and age. The indication for doing a blood culture was a suspicion of sepsis. Isolates were collected from Al Farwaniya hospital (which has pediatric and adult wards) from July 2013 until May 2016. The period of collection of isolates from Al Amiri hospital (which has neonatal, pediatric and adult wards) was from April 2013 until December 2015. The distance between the two hospitals is 26 km (16.2 miles). Al Farwaniya hospital is a 900-bed tertiary hospital which serves a population of 1.2 million people. Even though it serves both Kuwaitis and non-Kuwaitis, about 80% of the population served are non-Kuwaitis. Al Amiri hospital is a 418-bed tertiary hospital and it serves a population of 50,000 people. Although the hospital caters to both Kuwaitis and non-Kuwaitis, most of the people treated are Kuwaitis. All patients including diabetic patients and cancer patients have access to these hospitals, although for cancer treatment itself, there is a separate hospital. The patients included in our study were admitted in these hospitals, because of gastrointestinal symptoms and/or fever. Even though there are general guidelines for antimicrobial therapy, the antimicrobial therapy administered to patients in both hospitals was left to the discretion of attending physician. Antibiotics were given based on severity of infection, comorbidities and immune status of the patients.

Since typhoid is not endemic in Kuwait, typhoid vaccine is not offered to its residents. However, the vaccine is offered to residents travelling to high-risk areas.

**Blood culture.** Blood culture was done using BD BACTEC system (Becton Dickinson, MD, USA). From adults, 20 ml blood was drawn and 10 ml was inoculated into an aerobic bottle and 10 ml into an anaerobic bottle. From children, 1–5 ml of blood was drawn and inoculated into the aerobic bottle only. After inoculation of the bottle in the wards, they were immediately transported at ambient temperature to the hospital microbiology laboratory. Aerobic bottle signalling growth was subcultured on sheep blood agar, MacConkey agar and chocolate agar. The first two plates were incubated aerobically and the last plate in 5% CO2 atmosphere at 37°C for 24 h. Anaerobic bottle signaling growth was subcultured on brucella agar plate and the plate was incubated anaerobically at 37°C for 48 h. Identification of *Salmonella* and the initial antibiotic susceptibility were done from lactose non-fermenting colonies on MacConkey agar by Vitek 2 system (BioMerieux, Marcy l'Etoile, France). For grouping of *Salmonella*, the colony was inoculated into triple sugar iron agar and if the reaction was suggestive of *Salmonella*, the growth was used in a slide agglutination test against *Salmonella* polyvalent and group-specific antisera (Denka Seiken, Tokyo, Japan). As soon as *Salmonella* was detected in blood culture, empirical antimicrobial therapy for the patient was initiated. Further identification of *Salmonella* and antibiotic susceptibility testing were done as described below.

Stool was cultured for *Salmonella* from all *Salmonella* blood culture-positive patients. When warranted, urine culture was done. Stool was inoculated onto MacConkey agar and *Salmonella*-*Shigella* agar (SSA) and enriched in selenite F broth. Selenite F broth was subcultured onto SSA after 20 h of incubation. When urinary tract infection was suspected, urine was cultured on CLED (cysteine, lysine, electrolyte-deficient) agar and blood agar. All plates were incubated for 20–24 h at 37°C. Suspected *Salmonell*a colonies were subjected to identification as above. Subcultures of Salmonella isolates on MacConkey agar plates from the hospital microbiology laboratories were sent to the reference laboratory at the Faculty of Medicine, Kuwait University, where further tests, listed below, were arranged or carried out.

**Typing of *Salmonella* isolates.** Internal fragments of seven housekeeping genes (*thrA, purE, sucA, hisD, aroC, hemD, dnaN*) were amplified by PCR [8]. Sequences of the genes were trimmed to the required lengths (399–501 bps). Sequence types were assigned in accordance with the *Salmonella enterica* database, http://mlst.ucc.ie/mlst/dbs/Senterica. From sequence types, serovars were assigned according to the scheme of Achtman et al [9]. Isolates belonging to *S*. Tyhimurium and *S*. Enteritidis serovars were further confirmed by specific PCR assays [10].

**Antibiogram.** Antibiotic susceptibility test was carried out by E test (Biomerieux) and interpreted by the criteria laid down by Clinical and Laboratory Standards Institute (CLSI) [11]. The breakpoints (μg/ml) (intermediate resistance, resistance) for various antibiotics were as follows: ciprofloxacin (0.12 to 0.5, ≥1), chloramphenicol (16, ≥32), trimethoprim/sulfamethoxazole (not applicable, ≥4), gentamicin (8, ≥16), tetracycline (8, ≥16), ampicillin (16, ≥32), ceftazidime (8, ≥16), cefotaxime (2, ≥4), ceftriaxone (2, ≥4), imipenem (2, ≥4), meropenem (2, ≥4) and piperacillin/tazobactam (32 to 64, ≥128). Resistant and intermediate-resistant minimum inhibitory concentrations were categorized as resistant in accordance with clinical practice.

When there was a necessity of testing resistance to other antibiotics (streptomycin, neomycin, carbenicillin, trimethoprim and sulfonamide), this was done by Kirby-Bauer disc diffusion test [12].

Results were interpreted according to the criteria of CLSI [13]. *Escherichia coli* ATCC 25922 strain was used as a quality control strain.

### Extended spectrum β-lactamase (ESBL) production

**E test.** Isolates suspected of producing ESBL were tested for clavulanic-inhibitable ESBL production with E test ESBL strips–ESBL CT/CTL 16/1, ESBL TZ/TZL 32/4, ESBL PM/PML- as per manufacturer’s instructions (BioMerieux).

**Vitek 2 test.** The Vitek 2 ESBL test (bioMérieux) is based on the simultaneous assessment of the antibacterial activity of cefepime, cefotaxime and ceftazidime, measured either alone or in the presence of clavulanate. This test relies on card wells containing 1.0 mg/L of cefepime, or 0.5 mg/L of cefotaxime or ceftazidime, either alone or associated with 10 or 4 mg/L of clavulanate, respectively. After inoculation, cards were introduced into the Vitek 2 machine, and for each antibiotic tested, turbidity was measured at regular intervals. The proportional reduction of growth in wells containing a cephalosporin combined with clavulanate was then compared with that achieved by the cephalosporin alone and was interpreted as ESBL- positive or–negative through a computerized expert system (Advanced Expert System) [14].

**Detection of genes encoding ESBL.** PCR assays were performed to detect genes encoding *bla*_CTX-M_, *bla*_TEM_ and *bla*_SHV_ [7] and *bla*_PSE-1_ [15].

**AmpC disk test and modified three dimensional test (M3DT) for detection of AmpC β-latcamases.** This test was performed as described by Coskun et al [16]. Enhanced growth of bacteria around blank disks and AmpC disk or intersected growth in the zone of inhibition was considered positive.

**Pulsed-field gel electrophoresis (PFGE).** PFGE was performed according to the CDC protocol (http://www.cdc.gov/pulsenet/protocols.htm) using *XbaI* restriction enzyme (Roche, Mannheim, Germany) and a CHEF-DR III PFGE system (Bio-Rad, Munich, Germany). *Salmonella* Braenderup strain H9812(ATCC BAA 664) was used as a reference strain. The bands were visualized under UV light with Gel DOC(Bio-Rad) and analyzed with Bionumerics software (Applied Maths NV, Belgium). Pairwise similarities between patterns were calculated by DICE’s similarity coefficient. Clustering was based on unweighted pair-wise group method with averages (UPGMA) setting tolerance and optimization each at 1.5%.

**Whole genome sequencing (WGS).** Based on differences in banding patterns, *S*. Enteritidis and *S*. Typhimurium isolates were selected for whole genome sequencing. Sequencing libraries were prepared using the Nextera XT DNA sample preparation kit (Illumina, San Diego, CA, USA) and the sequence read data were produced on the Illumina NextSeq instrument (paired end, 150 base reads). Read data were submitted to the sequence read archive under project number PRJNA363099 (between 70x and 140x read depth coverage for each isolate). De novo assembly of the read data from each isolate was performed using MegaHit [17]. The resulting draft genome sequences were used to derive MLST (MLST:https://github.com/tseemann/mlst PubMLST: https://pubmlst.org/).

Abricate (https://github.com/tseemann/abricate) was used to detect virulence genes ([VFDB]: [18]). Antibiotic resistance gene profile was determined using Abricate and the Resfinder database [19].

**Data management and analysis.** Data on NTS bacteremia were obtained from laboratory records. Patient charts were traced, and relevant information was extracted. Results of bacterial analysis including serovars, antibiogram and WGS data were linked to patient data and tabulated in the reference laboratory. This dataset was used for analysis as appropriate.

**Statistics.** The difference in the prevalence of resistance to antibiotics in the two hospitals was calculated by Chi square test. A P value of ≤0.05 was considered significant.

**Ethical approval.** Bacterial isolates studied were a part of the routine collection at the Enteric Microbiology Reference Laboratory, Kuwait University, for further studies and archiving. It was not possible to get informed consent of patients as the clinical data were retrospectively collected. No additional specimens were collected for this study and patient identity was kept anonymous. Therefore, a waiver for informed consent, and approval for the study were granted by the Ethics Committee of Ministry of Health, State of Kuwait (permit number 898/2018).

## Results

**Fig 1:**
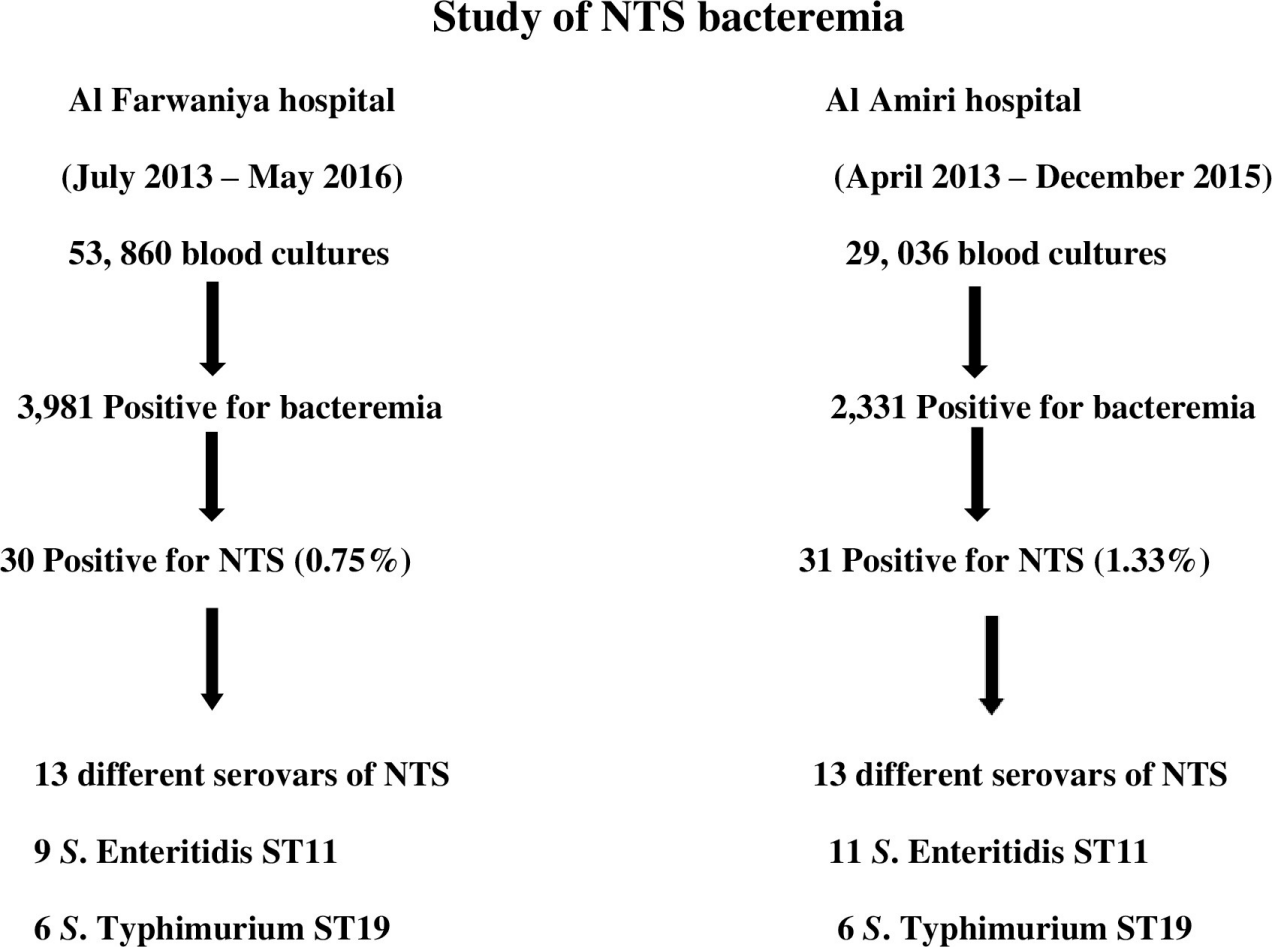
Non-typhoidal *Salmonella* isolation from blood cultures of patients in Al Farwaniya and Al Amiri hospitals. A fraction (0.75–1.33%) belonged to non-typhoidal *Salmonella* (NTS).

**NTS from blood culture.** The isolation of NTS from blood cultures of both hospitals is shown in Fig 1. In Al Farwaniya hospital, 53,860 blood cultures were done and 3981 were positive for microorganisms. Of the positive cultures, 30 were positive for a non-typhoidal *Salmonella* serovar (0.75%). There were 13 different *Salmonella* serovars, but 50% of them belonged to *S*. Enteritidis (all sequence type [ST]11) and *S*. Typhimurium (all ST19). In Al Amiri hospital, 29, 036 blood cultures were done and 2,331 were positive for microorganisms. Of the positive cultures, 31 were positive for a non-typhoidal serovar (1.33%). There were 13 different serovars of *Salmonella* infecting the patients, but 19 isolates (61.3%) belonged to *S*. Enteritidis (all ST11) and *S*. Typhimurium (all ST19).

Patient characteristics, *Salmonella* serovars isolated, response to therapy and patient outcome in Al Farwaniya and Al Amiri hospitals are presented in Table 1. Isolate numbers with suffix F are from Al Farwaniya hospital and those with suffix A are from Al Amiri hospital. Patients were both Kuwaitis and expatriates of many nationalities in both hospitals. Median age for all 61 patients was 58 y and 62.3% of patients were >50 y old. Eight patients (13.1%) were children <5 y old. Approximately 67% of patients had chronic diseases such as diabetes mellitus, cancer, blood disorder, kidney disease or lung disease. Forty patients (65.6%) had diarrhea and 26 of them (65%) had a *Salmonella* (identical to the corresponding blood isolate) cultured from the stool. Four of these 26 patients had also the corresponding *Salmonella* serovar cultured from the urine. One patient who had diarrhea did not have *Salmonella* cultured from the stool, but urine culture was positive for the corresponding blood isolate. None of the non-diarrheal patients had *Salmonella* cultured from the stool. Most patients responded to antibiotic therapy and were discharged. Many classes of antibiotics were used, but many received a cephalosporin. In addition, six patients (22A, 36A, 51A, 59A, 60A, 70A) received steroids. Only four patients died.

**Table 1:** Details of patients from Al Farwaniya hospital and Al Amiri hospital from whom *Salmonella* species were isolated from blood cultures.

Isolate No.	Age	Gender	Nationality	Diagnosis	*Salmonella* species from blood	Diarrhea present	Isolation of *Salmonella* from sites other than blood	Antibiotics given	Response to antibiotics	Outcome
1F	41	M	Indian	Abdominal pain	Livingston	No	No	Cefotaxime, Metronidazole	+ve	Discharged
2F	81	M	Kuwaiti	Cerebrovascular accident	Unspeciated type 2641	No	No	Ceftriaxone	Uncertain	Died
3F	38	F	Kuwaiti	Diarrhea	Enteritidis	Yes	No	Cefotaxime	+ve	Discharged
5F	58	M	Indian	Abdominal pain, fever, vomiting	Infantis	No	No	Piperacillin/ tazobactam	+ve	Discharged
7F	2.5	M	Kuwaiti	Intestinal obstruction, diarrhea	Enteritidis	Yes	No	Ceftriaxone	Uncertain	Transferred to surgical unit
9F	5	M	Saudi	Abdominal pain, sickle cell anemia	Typhimurium	No	No	Cefotaxime	+ve	Discharged
10F	27	M	Indian	Pancytopenia	Enteritidis	No	No	Ceftriaxone	Uncertain	Transferred to Infect Dis Hospital for? HIV infection
11F	<1	F	Egyptian	Fever, bronchiolitis, diarrhea	Typhimurium	Yes	No	Cefotaxime, gentamicin	+ve	Discharged
12 F	72	M	Kuwaiti	Diabetes mellitus, ischemic heart disease, diarrhea, vomiting, fever	Enteritidis	Yes	No	Piperacillin/ tazobactam, metronidazole	+ve	Discharged
13F	30	M	Filipino	Fever, diarrhea	Enteritidis	Yes	No	Cefotaxime, metronidazole	+ve	Discharged
14F	45	M	Egyptian	Diabetes mellitus, chronic renal failure, fever	Livingston	No	No	Cefotaxime	+ve	Discharged
15F	65	M	Pakistani	Diabetes mellitus, fever, jaundice	Livingston	No	No	Cefotaxime, clarithromycin	+ve	Discharged
16F	2	F	Kuwaiti	Diarrhea	Minnesota	Yes	No	None, oral rehydration only	+ve	Discharged
18F	56	M	Bangladeshi	Diabetes, eczema, diarrhea	Kentucky	Yes	No	Ciprofloxacin	+ve	Discharged
19F	54	F	Iraqi	Diabetes, diarrhea	Typhimurium	Yes	No	Ciprofloxacin, metronidazole	+ve	Discharged
46F	73	M	Kuwaiti	Diabetes mellitus, ischemic heart disease, fever, vomiting, diarrhea	Typhimurium	Yes	No	Meropenem	+ve	Discharged
47F	76	M	Kuwaiti	Diabetes mellitus, colon cancer, acute kidney injury, loss of appetite	Bareilly	Yes	No	Ceftriaxone	+ve	Discharged
48F	47	F	Kuwaiti	Sickle cell anemia, fever	Enteritidis	No	No	Ciprofloxacin	+ve	Discharged
52F	52	M	Egyptian	Colon cancer, weight loss	Typhimurium	No	No	Ciprofloxacin, amoxicillin/ clavulanic acid	+ve	Discharged
53F	70	F	Jordanian	Ischemic heart disease, chronic obstructive pulmonary disease, sinusitis	Enteritidis	No	No	Ceftriaxone	Uncertain	Transferred to neurosurgery for suspected brain abscess
55F	24	F	Kuwaiti	Fever, vomiting, diarrhea	Braenderup	Yes	Stool	None, oral rehydration only	+ve	Discharged
57F	47	F	Kuwaiti	Thalassemia, sickle cell anemia, upper respiratory infection, abdominal pain, chills	Poona	No	No	Ceftriaxone, azithromycin	+ve	Discharged
58F	46	M	Indian	Fever, thrombocytopenia, diarrhea	Telaviv	Yes	Stool	Piperacillin/ tazobactam, metronidazole	Uncertain	Discharged against recommendation
62F	65	M	Kuwaiti	Acute kidney injury, dehydration	Typhimurium	No	No	Meropenem, ceftriaxone	+ve	Discharged
64F	1	M	Kuwaiti	Diarrhea, fever	Enteritidis	Yes	Stool	Cefotaxime	+ve	Discharged
66F	59	M	Non- Kuwaiti*	Diabetes mellitus, end-stage renal disease, sepsis	Poona	No	No	No information	No information	No information
68F	1	M	Non- Kuwaiti*	Diarrhea	Anatum	Yes	Stool	None, oral rehydration only	+ve	Discharged
69F	66	M	Pakistani	Diabetes mellitus, cellulitis of thigh, diarrhea	Minnesota	Yes	Stool	Piperacillin/ tazobactam, cefotaxime, clindamycin	+ve	Discharged
71F	59	M	Indian	Hyponatremia, ruptured aortic valve	Enteritidis	No	No	Cefotaxime	-ve	Died
74F	1	M	Kuwaiti	Diarrhea	Uganda	Yes	No	None, oral rehydration only	+ve	Discharged
21A	79	F	Kuwaiti	Chronic kidney disease, liver chirrosis	Typhimurium	Yes	No	Ciprofloxacin	-ve	Died
22A	75	F	Kuwaiti	Diabetes mellitus, thrombocytopenia, Darriers disease	Enteritidis	No	No	Ceftriaxone	+ve	Discharged
23A	62	M	Syrian	Diabetes mellitus, ischemic heart disease	Enteritidis	Yes	Stool, urine	Ceftriaxone Metronidazole	+ve	Discharged
24A	54	M	Kuwaiti	Diabetes mellitus, Ormond's disease, periaertitis	Typhimurium	Yes	No	Ciprofloxacin Ceftriaxone	+ve	Discharged
25A	63	M	Kuwaiti	Acquired hemophilia	ST516	Yes	Stool	Ceftriaxone	+ve	Discharged
26A	70	F	Kuwaiti	Diabetes mellitus	Enteritidis	Yes	Stool	Ciprofloxacin	+ve	Discharged
27A	81	M	Kuwaiti	Diabetes mellitus, chronic kidney disease, liver cirrhosis	Enteritidis	Yes	Stool	Ceftriaxone, Meropenem	+ve	Discharged
28A	85	M	Kuwaiti	Chronic kidney disease, ischemic heart disease, dyslipidemia	ST27	Yes	Stool	Ceftriaxone	+ve	Discharged
29A	72	M	Kuwaiti	Diabetes mellitus	Typhimurium	Yes	Stool	Ceftriaxone	+ve	Discharged
30A	32	F	Kuwaiti	Ankylosing spondylitis, psoriasis	Enteritidis	No	No	Ceftriaxone Clindamycin	+ve	Discharged
31A	83	M	Kuwaiti	Diarrhea	New type	Yes	No	Ceftriaxone	+ve	Discharged
32A	33	F	Egyptian	Sickle cell anemia, hepatitis C virus infection	Enteritidis	Yes	Stool	No	Uncertain	Died within hours
33A	69	M	Kuwaiti	Diabetes mellitus	Enteritidis	Yes	Stool	Ciprofloxacin	+ve	Discharged
34A	65	F	Kuwaiti	Chronic kidney disease	Typhimurium	No	No	Ceftriaxone	+ve	Discharged
35A	53	M	Pakistani	Acute kidney injury	Agona	Yes	Stool	No	+ve	Discharged
36A	52	M	Iranian	Chronic autoimmune skin disease, sepsis, acute kidney injury	Typhimuriam	Yes	Stool	Ciprofloxacin Meropenem Metronidazole	+ve	Discharged
37A	69	M	Kuwaiti	Diabetes mellitus, chronic kidney disease	Anatum	Yes	Stool	Ciprofloxacin Meropenem	+ve	Discharged
38A	2	M	Kuwaiti	Diarrhea	Enteritidis	Yes	Stool	Ceftriaxone	+ve	Discharged
39A	73	M	Kuwaiti	Diabetes mellitus, skin disease, acute renal injury, sepsis	Typhimurium	No	No	Ceftriaxone Clindamycin	+ve	Discharged
40A	54	M	Indian	Diabetes mellitus, ulcerative colitis	Enteritidis	Yes	Stool	Ciprofloxacin	+ve	Discharged
41A	80	F	Kuwaiti	Ischemic heart disease, toxic megacolon, dyslipidemia	Albany	Yes	Stool	Ceftriaxone	+ve	Discharged
42A	58	F	Kuwaiti	Diabetes mellitus, chronic kidney disease	Typhimurium	Yes	Stool	Ceftriaxone	+ve	Discharged
43A	73	M	Kuwaiti	Diabetes mellitus, dyslipidemia	Newport	Yes	Stool	Ciprofloxacin	+ve	Discharged
44A	75	M	Kuwaiti	Liver cirrhosis	Enteritidis	Yes	Stool	Ciprofloxacin Ceftriaxone	+ve	Discharged
45A	70	M	Kuwaiti	Diabetes mellitus, chronic kidney disease, fever	ST324/ST2606	No	No	Ciprofloxacin	+ve	Discharged
49A	60	F	Kuwaiti	Chronic kidney disease	Minnesota	Yes	Stool, urine	Ciprofloxacin Ceftriaxone	+ve	Discharged
51A	45	M	Kuwaiti	Diabetes mellitus, Addison's disease, asthma	Typhimurium	No	Stool, urine	Ceftriaxone Clarithromycin	+ve	Discharged
59A	36	M	Kuwaiti	Endstage renal disease	Bareilly	Yes	Stool, urine	Ciprofloxacin	+ve	Discharged
60A	30	M	Kuwaiti	Lupus nephritis	Enteritidis	Yes	Urine	Ciprofloxacin Metronidazole Amphotericin B	+ve	Discharged
61A	78	F	Kuwaiti	Diabetes mellitus, dyslipidemia, pneumonia, vomiting	Give	No	No	Ciprofloxacin Cefotaxime	+ve	Discharged
70A	36	F	Kuwaiti	Multiple sclerosis	Typhimurium	Yes	No	piperacillin/ Tazobactam	+ve	Discharged

*Non-Kuwaiti, but nationality uncertain

**Antibiogram.** The prevalence of resistance to all antibiotics was similar in both hospitals (P>0.05 for all comparisons) (S1A & S1B Table). Therefore, resistance for isolates from both hospitals was combined and is presented in Table 2. The prevalence of resistance to ampicillin, ciprofloxacin and tetracycline ranged between 36.1 to 50.8%. The prevalence of resistance to other antibiotics was either negligible or absent.

**Table 2:** Antibiotic susceptibility of non-typhoidal *Salmonella* isolated from blood cultures of 61 patients admitted in Al Farwaniya and Al Amiri hospitals, Kuwait.

Antibiotic	Range (μg/ml)	MIC_50_	MIC_90_	No. (%) resistant
Ampicillin (AMP)	0.125 - ›256	2	›256	22 (36.1)
Ceftazidime (CAZ)	0.094–32	0.38	2	5 (8.2)
Cefotaxime (CTX)	0.094–128	0.125	0.5	6 (9.8)
Ceftriaxone (CRO)	0.032–24	0.094	1.5	5 (8.2)
Imipenem (IPM)	0.032–1.5	0.19	0.5	0 (0.0)
Meropenem(MEM)	0.004–0.38	0.032	0.125	0 (0.0)
Piperacillin/Tazobactam (TZP)	1.0–8	2	4	0 (0.0)
Tetracycline (TET)	1.5–256	8	256	31 (50.8)
Gentamicin (GM)	0.38–48	0.5	0.75	2 (3.3)
Trimethoprim—Sulfamethoxazole (SXT)	0.047–32	0.25	32	9 (14.8)
Chloramphenicol (CHL)	0.032–256	6	16	7 (11.5)
Ciprofloxacin (CIP)	0.016–1.5	0.047	0.5	24 (39.3)

The resistance phenotypes in both hospitals are shown in Table 3. A total of 52 of 61 isolates (85. 2%) were resistant to one or more antibiotics tested. Of the resistant isolates, 30(60%) were either *S*. Typhimurium or *S*. Enteritidis. Among the 16 multi-resistant (resistant to three or more classes of antibiotics) isolates, 8 (50%) were either *S*. Typhimurium or *S*. Enteritidis. Fourteen isolates (23%) were resistant to a cephalosporin.

**Table 3:** Resistance phenotypes of non-typhoidal *Salmonella* isolates.

Resistance phenotype	Number of isolates
Cip	1
Amp	2
Tet	16
Cip, Tet	7
Amp, Tet	3
Tet, Chl	2
Tet, Cro	1
Tet, Ctx	1
Cip, Sxt, Tet	2
Cip, Tet, Ctx	1
Cip, Chl, Tet	1
Amp, Caz, Ctx	2
Amp, Sxt, Tet	2
Amp, Tet, Caz	1
Amp, Ctx, Cro	1
Cip, Amp, Tet	1
Cip, Amp, Chl, Tet	1
Chl, Tet, Caz, Ctx, Cro	1
Cip, Amp, Chl, Sxt, Gent, Tet	1
Cip, Amp, Chl, Sxt, Tet, Caz	1
Cip, Chl, Tet, Caz, Ctx, Cro	1
Cip, Amp, Chl, Tet, Caz, Ctx, Cro	2
Cip, Amp, Chl, Sxt, Gent, Tet, Ctx, Cro	1

Cip: Ciprofloxacin, Amp: Ampicillin, Chl:Chloramphenicol, Cro: Ceftriaxone, Ctx: Cefotaxime

Sxt = Trimethoprim-sulphamethoxazole, Gen = Gentamicin, Tet = Tetracycline, Caz = Ceftazidime

**ESBL production.** One isolate (69F) from Al Farwaniya hospital was resistant to all three cephalosporins tested. Two isolates (45A and 49A) from Al Amiri hospital were resistant to all three cephalosporins. All three isolates were negative for clavulanic acid-inhibitable ESBL production by E test and for specific genes encoding ESBL, but were positive for ESBL production by both Vitek 2 test and E test. Two isolates (69F and 49A) were positive for AmpC test.

**Fig 2:**
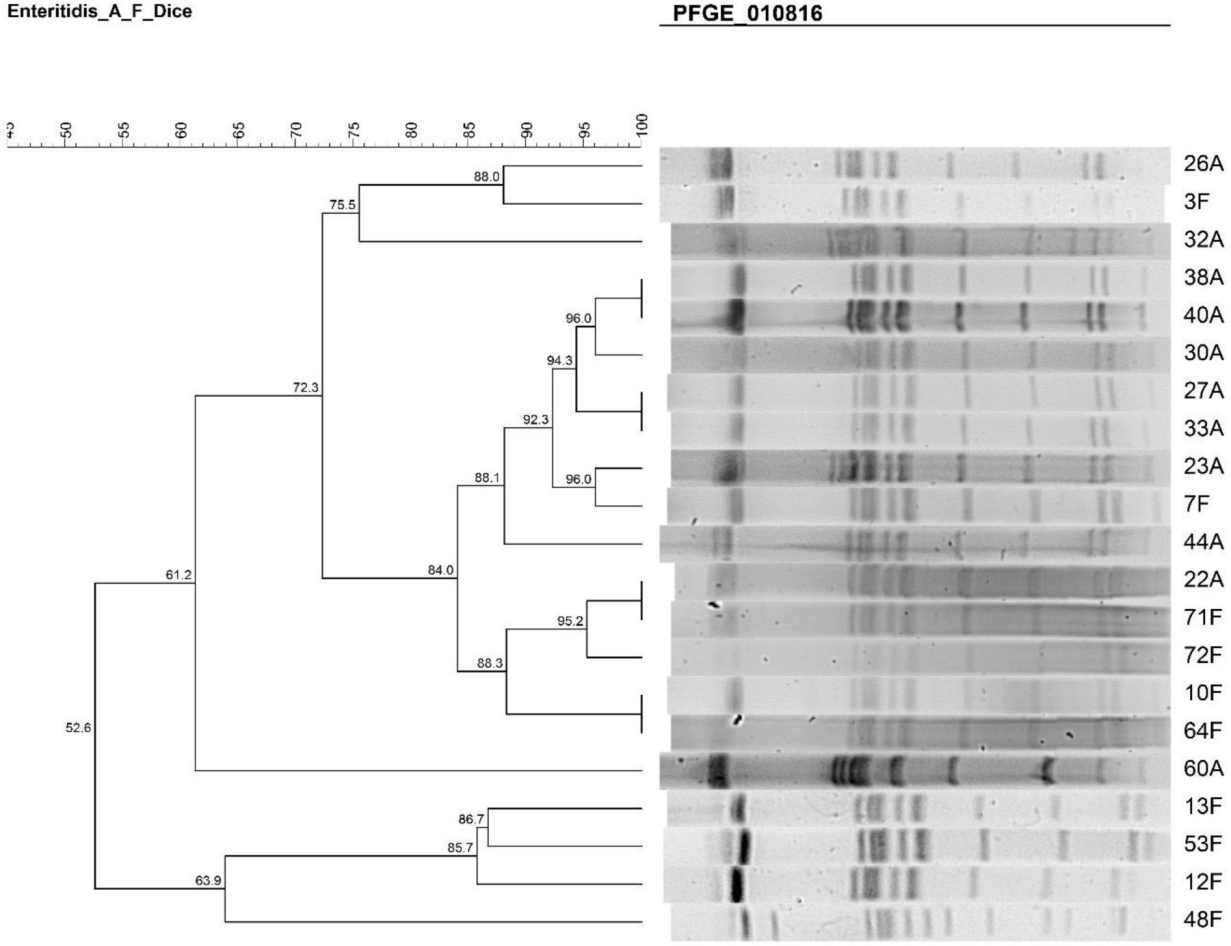
PFGE dendrogram showing the relationship among *S*. Enteritidis. The similarities between isolates were evaluated using Dice coefficient and UPGMA clustering method.

**PFGE.** The dendrogram of *S*. Enteritidis isolates from both Al Amiri and Al Farwaniya hospitals is shown in Fig 2. There were three clusters–cluster 1 comprised isolates 12F, 53F, and 13F; cluster 2 comprised isolates 64F, 10F, 72F, 71F, 22A, 44A, 7F, 23A, 33A, 27A, 30A, 40A, and 38A; and cluster 3 comprised isolates 32A, 3F and 26A. Isolates 48F and 60A were outliers.

**Fig 3:**
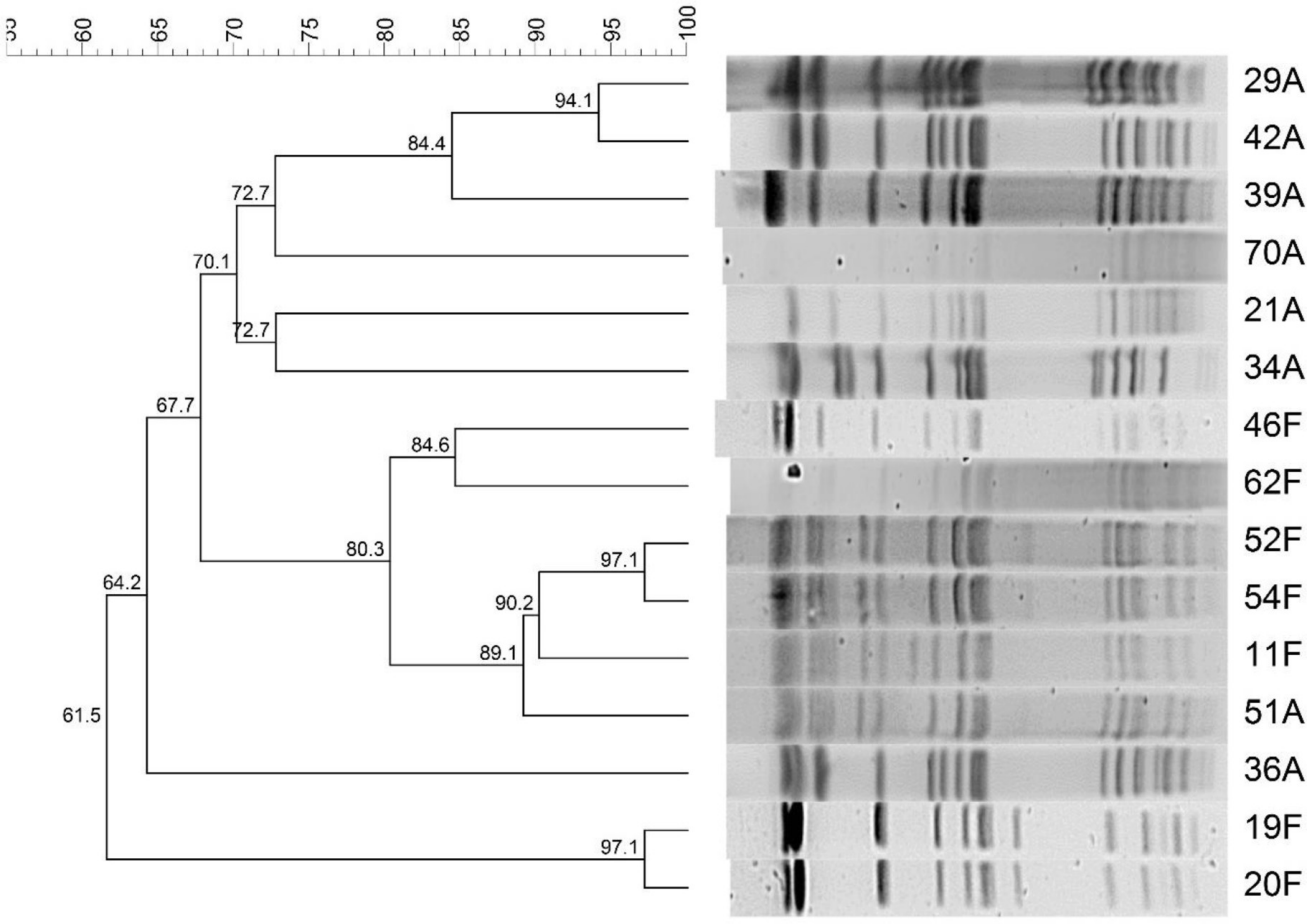
PFGE dendrogram showing the relationship among *S*. Typhimurium. The similarities between isolates were evaluated using Dice coefficient and UPGMA clustering method.

The dendrogram of *S*. Typhimurium isolates from both Al Amiri and Al Farwaniya hospitals is shown in **Fig 3.** There were three clusters–cluster 1 comprised of isolates 20F and 19F; cluster 2 comprised isolate 36A; cluster 3 comprised isolates 51A, 11F, 54F, 52F, 62F, and 46F; cluster 4 comprised isolates 34A, 21A, 70A, 39A, 42A and 29A. Isolate 36A was an outlier.

**WGS.** Four *S*. Enteritidis isolates from Al Amiri hospital (23A, 32A, 38A, 60A) and two *S*. Enteritidis isolates from Al Farwaniya hospital (12F, 48F) were sequenced. Five *S*. Typhimurium isolates from Al Amiri hospital (21A, 51A, 34A, 29A, 36A) and four *S*. Typhimurium isolates from Al Farwaniya hospital (46F, 52F, 11F, 19F) were sequenced. These isolates belonged to different clusters by PFGE.

**Fig 4:**
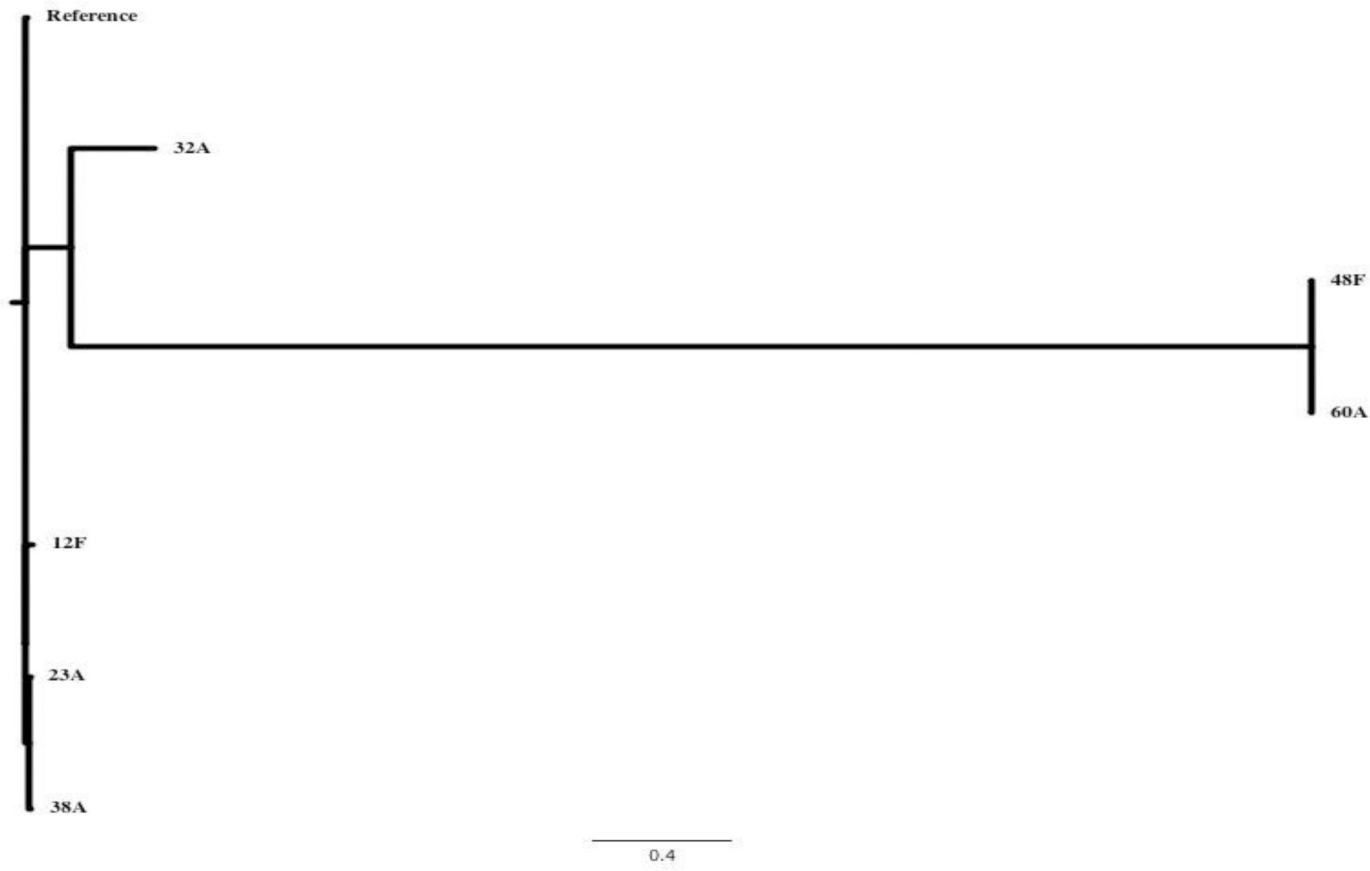
An unrooted tree showing the inferred relationship between the six S. Enteritidis clinical isolates. The *S*. Enteritidis strain P125109 was used as the reference genome sequence for read mapping. More than 97% of the reference genome was included in the core genome derived for this set of isolates. The core genome contained 1,999 sites that varied in one or more of the clinical isolates. The tree was inferred using Fast Tree and the greatest pairwise distance between isolates is 1670 SNPs, e.g. between isolates 60A and 12F.

The phylogenetic relationship among the six *S*. Enteritidis isolates based on core genome sequence is shown in **Fig 4.** The genome of *S*. Enteritidis strain P125109 was used as the reference genome sequence. More than 97% of the reference genome was present in the genomes of the six clinical *S*. Enteritidis isolates. The core genome contained 1999 sites that varied in the six clinical isolates. The pairwise distance was greatest (1670 SNPs [single nucleotide polymorphisms]) between isolates 60A and12F. There were three clusters formed by 12F, 23A & 38A; 32A; and 48F & 60A.

The phylogenetic relationship based on the core genomes of the six clinical *S*. Enteritidis isolates in relation to core genomes of 59 *S*. Enteritidis strains from different parts of the world for which there are closed genome sequences, is shown in **S1 Fig.** The greatest pairwise distance of 1833 SNPs was found between isolate 48F and OLF-SE9-10012, a clam isolate of 2010 from Canada. There were a total of seven clusters and Kuwaiti isolates exhibited three clusters: 12F, 23A & 38A; 32A; and 48F & 60A. The details of genomes of 60 *S*. Enteritidis strains which were used for comparative analysis are given in S2 Table.

**Fig 5:**
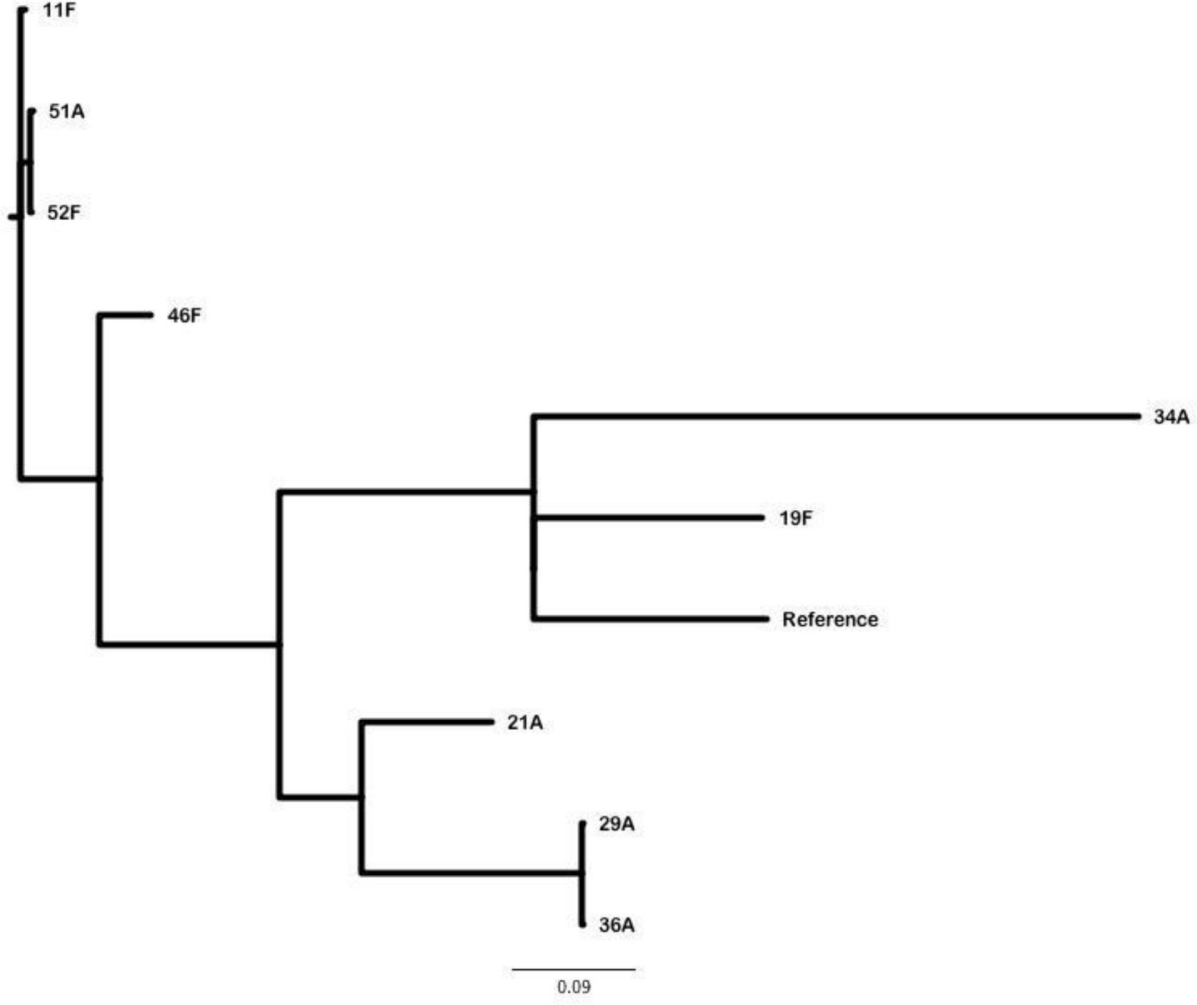
An unrooted tree showing the inferred relationship between the nine *S*. Typhimurium clinical isolates. The *S*. Typhimurium strain LT2 was used as the reference genome sequence for read mapping. More than 95% of the reference genome was included in the core genome derived for this set of isolates. The core genome contained 1,979 sites that varied in one or more of the clinical isolates. The tree was inferred using Fast Tree and the greatest pairwise distance between isolates is 1,017 SNPs between isolates 36A and 34A.

The phylogenetic relationship of the nine *S*. Typhimurium isolates based on their core genome sequence is shown in **Fig 5.** The genome of *S*. Typhimurium strain LT2 was used as the reference genome. More than 95% of the genome of the reference strain was contained in the genomes of the nine clinical *S*. Typhimurium isolates. The core genome contained 1979 sites that varied among the isolates. The greatest pairwise distance of 1017 SNPs was found between isolates 36A and 34A. Kuwaiti isolates formed five clusters: 11F, 51A &52F; 46F; 34A & 19F; 21A; 29A & 36A.

The phylogenetic relationship of the nine *S*. Typhimurium isolates based on their core genomes in relation to 21 *S*. Typhimurium strains from different parts of the world whose closed genome sequences are known, is shown in **S2 Fig.** The greatest pairwise distance of 1333 SNPs was found between isolates 22792 (a cormorant isolate of 2008 from Canada) and RM10961 (an isolate from an agricultural produce in the USA, whose isolation details are not known). There were a total of 10 clusters and Kuwait isolates formed four clusters: 19F; 34A; 21A, 29A & 36A; 46F, 11F, 51A & 52F. The details of genomes of 21 *S*. Typhimurium strains which were used for comparative analysis are shown in S2 Table.

**Virulence genes.** The catalog of virulence genes identified in the whole genome-sequenced *S*. Enteritidis and *S*. Typhimurium is shown in S3 Table. The organisms possessed genes encoding a variety of type III secretion system proteins that manipulate host cell transduction pathways and cellular processes to pathogen’s advantage. Other genes included were those for chemotaxis and different fimbriae; anti- inflammatory effector genes for enhancing colonization; genes for resistance to antimicrobial peptides, mouse cecal colonization and prolonged shedding, cellular invasion, enteritis, fluid secretion etc. Of note are: *ste* gene (for spread and survival in host tissue), *sse* gene (for proliferation inside the macrophage and systemic infection in mice), *sodC1* gene (for resistance to phagocytosis), *rck* gene (for serum resistance) and *spv* genes (for proliferation inside the macrophages and late apoptosis and spread of infection).

**Antibiotic resistance genes.** Antimicrobial resistance genes were found in some of the whole genome- sequenced *S*. Typhimurium and *S*. Enteritidis isolates. These are shown in Table 4. Based on the presence of an antimicrobial resistance gene, resistance to the corresponding antibiotic was found in the isolate as indicated in the footnote to the Table. Even though *S*. Typhimurium isolate 34 A possessed *bla*_*CARB-2*_ gene, it was susceptible to imipenem and meropenem. *S*. Typhimurium isolate 34A resistant to ampicillin, chloramphenicol and tetracycline had corresponding resistance genes. The same isolate was ciprofloxacin- intermediate resistant and had a substitution of asparagine (N) for aspartic acid (D) at position 87 in the *gyrA* gene. Multi-resistant *S*. Typhimurium isolate 51A was ciprofloxacin-intermediate resistant and had a substitution of tyrosine (Y) for serine (S) at position 83 in the *gyrA* gene [20]. *S*. Enteritidis isolates, 23A and 38A carried the ESBL gene, *bla*_TEM-1B_ and were resistant to ampicillin [21]. Cephalosporin-intermediate-resistant isolates were not among the isolates that were subjected to whole genome sequencing.

**Table 4:** Antibiotic resistance genes in sequenced *S*. Enteritidis and *S*. Typhimurium isolates.

Gene^a^	*S*. Enteritidis Isolates						*S*. Typhimurium Isolates									GenBank	Resistance
	12F	23A	32A	38A	48F	60A	11F	19F	21A	29A	34A	36A	46F	51A	52F		
***aac(6')-Iaa_1***	100^b^	100	100	100	100	100	100	100	100	100	100	100	100	100	100	NC_003197	Aminoglycoside resistance
***aph(3'')-Ib_5***	-^c^	-	-	-	-	-	-	-	100	-	-	-	98.1	100	100	AF321551	Aminoglycoside resistance
***aph(3')-Ia_1***	-	-	-	-	-	-	-	-	100	-	-	-	-	-	-	V00359	Aminoglycoside resistance
***aph(6)-Id_1***	-	-	-	-	-	-	-	-	100	-	-	-	100	100	100	M28829	Aminoglycoside resistance
***blaCARB-2_1*** ^**d**^	-	-	-	-	-	-	-	-	-	-	100	-	-	-	-	M69058	Beta-lactam resistance
***blaTEM-1B_1*** ^**e**^	-	100	-	100	-	-	-	-	100	-	-	-	-	-	-	JF910132	Beta-lactam resistance
***dfrA5_1***	-	-	-	-	-	-	-	-	100	-	-	-	-	-	-	X12868	Trimethoprim resistance
***floR_2***	-	-	-	-	-	-	-	-	-	-	100	-	-	-	-	AF118107	Phenicol resistance
***sul2_2***	-	-	-	-	-	-	-	-	100	-	-	-	100	100	100	AY034138	Sulphonamide resistance
***tet(A)_6***	-	-	-	-	-	-	-	-	97.8	-	-	-	100	97.8	97.8	AF534183	Tetracycline resistance
***tet(G)_2***	-	-	-	-	-	-	-	-	-	-	100	-	-	-	-	AF133140	Tetracycline resistance
***gyrA* mutation**	-	-	-	-	-	-	-	-	-	-	D87N	-	-	S83Y	-	X78977	Fluoroquinolone resistance

^a^Gene symbol as per ResFinder database

^b^Percent gene coverage

^c^Negative for presence

^d^Alternate name: PSE-1, *blaP1b*

^e^Alternate name: Rbla TEM-1

Resistance to trimethoprim, sulphonamide and carbenicillin was tested by disk diffusion method. Resistance to other antibiotics was tested by E test

## Discussion

A small fraction (0.75–1.33%) of blood culture-positive isolates only accounted for NTS bacteremia in Kuwait. The prevalence of NTS bacteremia as a proportion of community-acquired bacteremia varies widely according to geographic areas. It was 8% in Southern Africa, 25% in Central Africa, 27% in East Africa, and 18% in Western Africa [22]. In a multicenter study covering Indonesia, Thailand and Vietnam, the prevalence of NTS in individuals positive for any blood-stream bacterial infection was 27.5% among children, and 11.7% among adults [23]. In a study in Bangladesh, the prevalence of NTS in blood culture was 0.16% [24]. A Malaysian study found a prevalence rate of 16.2% with most of the cases occurring in children below 1 y of age [25]. The risk factors contributing to NTS bacteremia are extremes of age, immunosuppressive therapy, and underlying comorbidities such as diabetes mellitus, cancer, cardiovascular diseases etc. that affect the immune system [26]. In Africa, the risk factors for children are: sickle cell disease and malnutrition, and the risk factors not associated with age are malaria, anemia and human immunodeficiency virus infection [22, 26–32]. In Kuwait too, the affected patients were either old people or children. The patients also suffered from comorbidities such as diabetes mellitus, cancer, blood disorders, lung diseases and kidney diseases. In our case series, 4 out of 61 patients died with a case- fatality rate (CFR) of 6.6%. This low CFR in our study may be attributed to prompt and appropriate therapy of our patients including better management of underlying diseases. CFR was 20.6% in Sub-Saharan Africa [22]; 25% in Bangladesh [24]; 33% in Israel [33]; and 8.7% in Taiwan [34]. A higher CFR of 40.5% was seen among NTS bacteremic patients with malignancy compared to 17.7% among NTS bacteremic patients without malignancy in Taiwan [35]. In Al Farwaniya hospital, 16 patients (53.3%) had diarrhea, and of these, 5 patients (31.3%) had the same serovar of *Salmonella* as the one in blood culture isolated from the stool. There was an interval of 1–2 days between blood culture and stool culture. If blood culture of a patient was positive for a NTS, the patient was empirically treated with antibiotics during this interval. This antibiotic treatment would have affected the recovery of *Salmonella* in the stool culture of the patient. Approximately 5% of individuals with gastrointestinal illness caused by NTS will develop bacteremia [36]. However, in primary NTS bacteremia, most of the patients do not develop diarrhea and NTS is also not cultured from stool [37]. This suggested that a higher proportion of Kuwaiti patients with NTS bacteremia had gastrointestinal illness with the isolation of corresponding blood isolates from stools. Many different NTS serovars caused blood stream infection in Kuwaiti patients. However, 50–60% of the infections were due to *S*. Enteritidis and *S*. Typhimurium species in the two hospitals. Nonetheless, in Al Farwaniya hospital, most of the expatriate patients from South Asia and Southeast Asia had infection with neither of these serovars.

Although many serovars of NTS can cause blood stream infection, *S*. Enteritidis and *S*. Typhimurium serovars are the predominant serovars causing blood stream infection in many parts of the world [22, 29, 38–40]. NTS infection is usually zoonotic in origin contracted by contact with animals or consumption of contaminated water or food of animal origin [41]. However, there is also evidence of person- to- person transmission [42, 43]. In the largely urban environment, coupled with the cultural context of Kuwait where pet animals such as dogs are a religious taboo, contact with animals is less likely, and the most likely routes are consumption of contaminated food and person- to- person contact.

Patients were treated with many antibiotic classes, but most were treated with a third-generation cephalosporin–ceftriaxone, cefotaxime or ceftazidime. Most patients responded to these antibiotics. The antibiotic response concurs with the susceptibility data (Tables 2 and 3). ESBL production has been reported in NTS worldwide including in Kuwait [7, 44, 45]. In the current study, three out of 13 cephalosporin- resistant isolates showed ESBL production. In two isolates, ESBL production may be mediated by AmpC. All the 13 resistant isolates were negative for specific genes. Resistance may also be due to other mechanisms such as an altered porin with decreased entry of the antibiotic into bacterial cell, with the bacteria showing intermediate susceptibility [46, 47]. A combination of tests needs to be done for the detection of ESBL production [48]. Isolate 34A possessed the carbapenemase resistant gene, *bla*_*CARB-2_1*_, yet, it was susceptible to carbapenem. This concurs with a previous report [15]. Most of the isolates were resistant to one or more antibiotics, and 26.2% were multidrug-resistant. Half of the multidrug-resistant isolates belonged to *S*. Typhimurium or *S*. Enteritidis. Drug resistance is a problem among NTS isolates in many parts of the world [39, 27, 28, 49, 50, 51, 52]. Studies in African countries showed a higher prevalence of multidrug-resistant bacteria (40 to 100%) [28, 49–52] than in our study.

We typed all NTS isolates by PFGE and some selected isolates by WGS. There were discrepancies between the two typing methods. This is not unexpected as approaches to typing are different in the two methods. Nevertheless, studies have shown that WGS is more discriminatory than traditional typing methods including PFGE [53,54]. Moreover, WGS gives a near thorough sequence information of all genes present in the bacteria. In our series, the MLST of *S*. Typhimurium was ST19 and that of *S*. Enteritidis was ST11.

In Sub- Saharan Africa, the predominant ST of *S*. Typhimurium was ST313 [55, 56]. ST 19 was the predominant type found in both Europe and North America [57]. ST11 and ST19 were the predominant types causing gastroenteritis in Qatar, another Middle Eastern country [58]. ST19 was the predominant type in Iran being isolated from blood, urine and stool specimens [59]. It was also the predominant ST found in China [60]. *S*. Enteritidis ST11 causes diarrhea and blood stream infection world-wide. It caused blood stream infections from Mozambique [61], Kenya [55], and Vietnam [62].

Sequencing of our NTS isolates showed that they carried a full complement of virulence genes. Of note are the presence of genes that contribute to systemic spread and survival in the blood stream—*ste* gene for spread and survival in the host tissue through multiplication in a membrane-bound compartment, SCV [63], *sse* gene for survival and replication inside the macrophages via a type III secretory system[64], *sodC1* gene for protection of bacteria against superoxide generated within phagocytes [65], *rck* gene for serum resistance [66], and *spv* genes for virulence of NTS to cause extra-intestinal disease by cytotoxicity and apoptosis of macrophages [67].

Thus, our data showed that unlike in sub-Saharan Africa and some parts of Asia, there was only a low-case fraction of NTS isolated from blood cultures done at these two hospitals in Kuwait. From a public health point of view, these patients need to be protected from contracting NTS infection by provision of thoroughly cooked foods of animal origin, and a high standard of personal hygiene of caregivers. A significant proportion of our patients had gastrointestinal illness, and mortality was negligible. Similarities with other studies included the following: the patients affected were young or old; most patients had immunocompromising co- morbidities; an array of serovars of *Salmonella* caused blood stream infection, but most of the isolates were *S*. Typhimurium and *S*. Enteritidis; and drug resistance was a problem in the isolates, but most infections were treated with a third-generation cephalosporin with or without other antibiotics. Unlike other studies, we performed phylogenetic analysis of all *S*. Typhimurium or *S*. Enteritidis isolates by PFGE and some selected PFGE typed isolates by WGS. These two typing methods showed that the isolates showed closely related clusters. Phylogeny by core genome analysis showed a close relationship with *S*. Typhimurium and *S*. Enteritidis from other parts of the world. WGS showed that *S*. Typhimurium and *S*. Enteritidis had a complement of virulence genes mediating extra-intestinal infection and antibiotic resistance genes mostly corresponding to the resistance phenotypes.

Our study has several limitations since it is a hospital-based study and cases were diagnosed by blood culture. For hospitalization, there is a selection bias towards more severe cases and associated conditions. Hospitalized cases are not truly representative of community cases where a spectrum of severity of cases can occur, and because of this, community population at risk cannot be properly defined. Therefore, our findings cannot be extrapolated to a general population. Hospital records are not primarily designed for research purposes because of incomplete and unstandardized information and diagnostic variability between hospitals. Retrospective studies may have inferior level of evidence compared with prospective studies, and may be subject to confounding variables that may be present, but not measured. Moreover, temporal relationships are difficult to assess in retrospective studies. The concentration of NTS in blood is about 1 cfu per ml [68]. Therefore, conventional blood culture and even polymerase chain reaction (PCR) method may not be sensitive enough to detect all true positive cases [69]. This is prompting investigators to design more sensitive methods [70].

## Supporting information

S1 Fig An unrooted tree showing the inferred relationship between the six *S.* Enteritidis clinical isolates and 59 *S.* Enteritidis strains for which there is closed genome sequence. (TIF)

S2 Fig An unrooted tree showing the inferred relationship between the nine *S.* Typhimurium clinical isolates and 21 *S.* Typhimurium strains for which there is closed genome sequence. (TIF)

S1 Table **A. Prevalence of antimicrobial resistance in non-typhoidal *Salmonella* from blood cultures of 30 patients in Al Farwaniya hospital. B. Prevalence of antimicrobial resistance in non-typhoidal *Salmonella* from blood cultures of 31 patients in Al Amiri hospital**.
(DOCX)

S2 Table *S. enterica* strains with complete genome sequences used for core genome comparisons. (XLSX)

S3 Table *S. enterica* virulence genes detected in the genome sequences of the *S.* Enteritidis and *S.* Typhimurium isolates. (XLSX)
