# Supplementary figures and images for: Non-typhoidal Salmonella blood stream infection in Kuwait: Clinical and microbiological characteristics
Source: PLoS Negl Trop Dis. 2019 Apr 15;13(4):e0007293. doi: 10.1371/journal.pntd.0007293 (PMC6483562; doi:10.1371/journal.pntd.0007293)

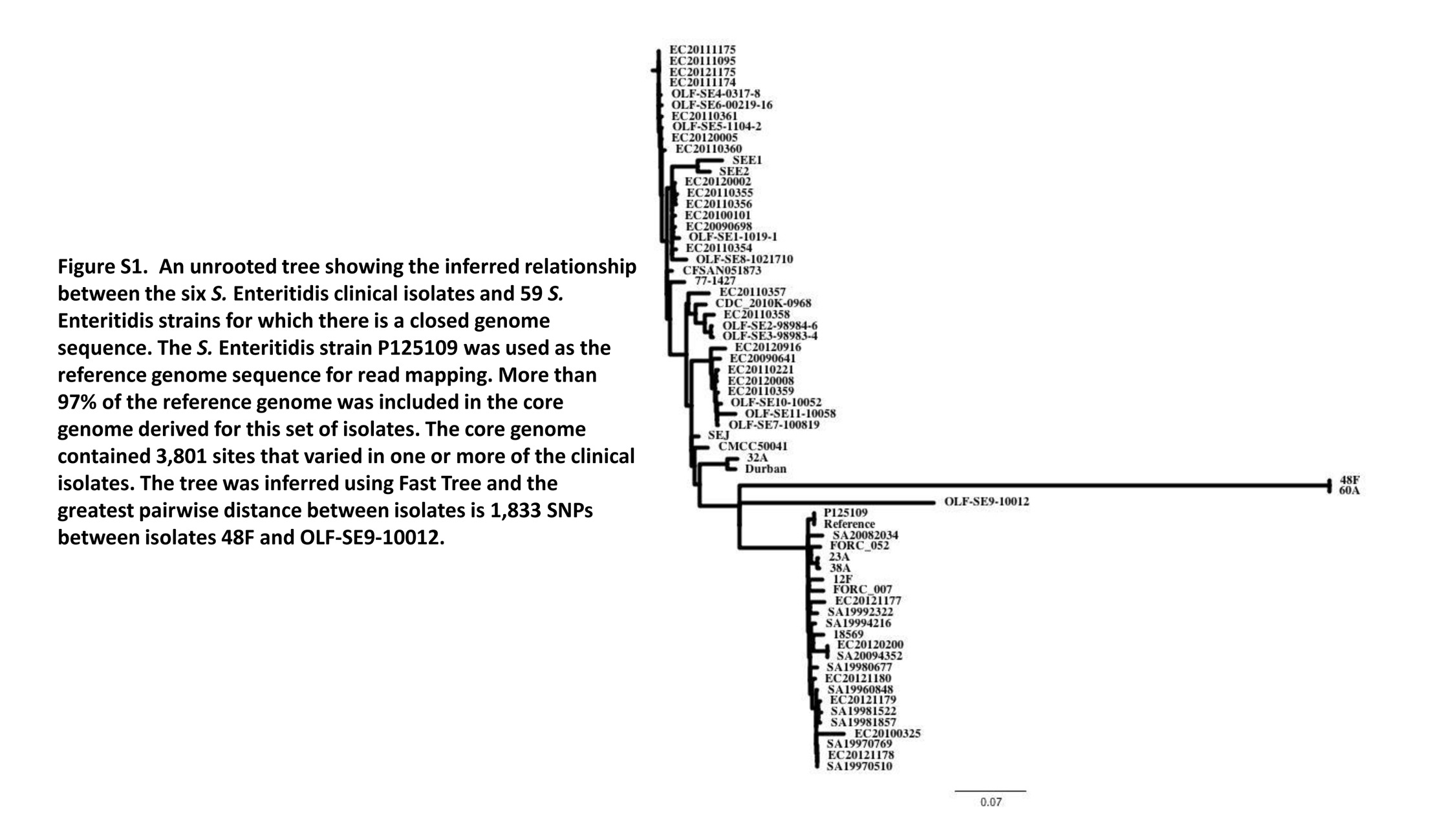

Supplement: S1 Fig — (TIF) [file pntd.0007293.s001.tif]

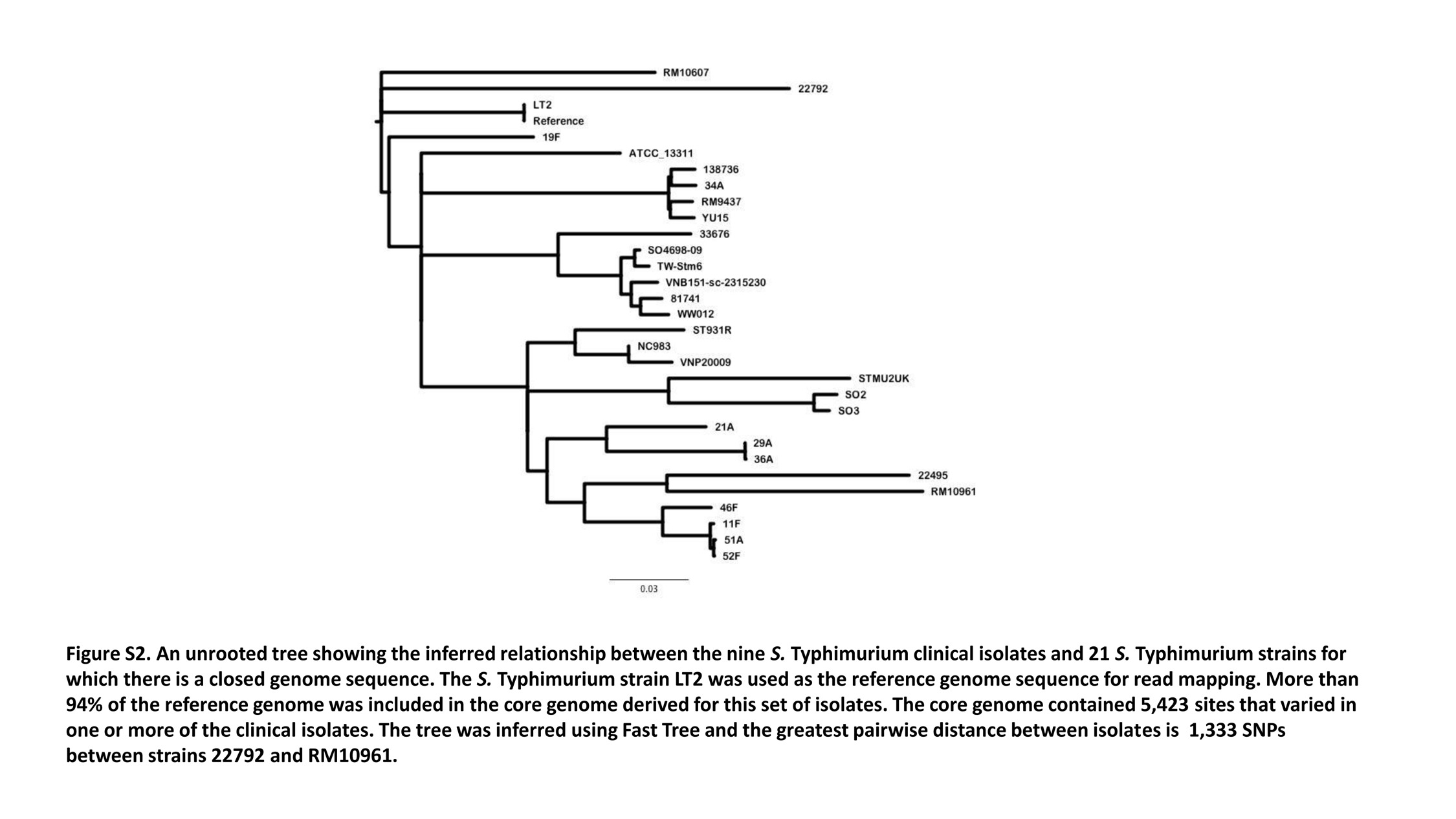

Supplement: S2 Fig — (TIF) [file pntd.0007293.s002.tif]
