# Supplementary material for: Non-typhoidal Salmonella blood stream infection in Kuwait: Clinical and microbiological characteristics
Source: PLoS Negl Trop Dis. 2019 Apr 15;13(4):e0007293. doi: 10.1371/journal.pntd.0007293 (PMC6483562; doi:10.1371/journal.pntd.0007293)
Supplement: S1 Table — A. Prevalence of antimicrobial resistance in non-typhoidal Salmonella from blood cultures of 30 patients in Al Farwaniya hospital. B. Prevalence of antimicrobial resistance in non-typhoidal Salmonella from blood cultures of 31 patients in Al Amiri hospital. (DOCX) [file pntd.0007293.s003.docx]

| Table S1A. Antibiotic Susceptibility of non-typhoidal *Salmonella* isolated from blood culture of 30 patients admitted in Al Farwaniya hospital Kuwait | | | | |
| --- | --- | --- | --- | --- |
| Antibiotic | Range(µg/ml) | MIC 50 | MIC90 | No. (%) resistant |
| Ampicillin (AMP) | 0.125 - ›256 | 2 | ›256 | 11 (36.6) |
| Ceftazidime (CAZ) | 0.094 - 32 | 0.25 | 2 | 3 (10.0) |
| Cefotaxime (CTX) | 0.94 - 128 | 0.19 | 4 | 4 (13.3) |
| Ceftriaxone (CRO) | 0.032 - 24 | 0.125 | 1.5 | 3 (10.0) |
| Imipenem (IPM) | 0.094 – 1.5 | 0.25 | 1.0 | 0 (0) |
| Meropenem(MEM) | 0.004 – 0.38 | 0.032 | 0.125 | 0 (0) |
| Piperacillin/Tazobactam (TZP) | 1.5 - 6 | 2 | 4 | 0 (0) |
| Tetracycline (TET) | 1.5 - 256 | 8 | 256 | 16 (53.3) |
| Gentamicin (GM) | 0.38 - 48 | 0.5 | 1 | 1(3.3) |
| Trimethoprim - Sulfamethoxazole (SXT) | 0.125 - 32 | 0.25 | 2 | 5(16.6) |
| Chloramphenicol (CHL) | 0.032 - 256 | 6 | 256 | 5 (16.6) |
| Ciprofloxacin (CIP) | 0.016 - 1.5 | 0.19 | 0.75 | 16 (53.3) |

| Table S1B. Antibiotic Susceptibility of non-typhoidal *Salmonella* isolated from blood culture of 31 patients admitted in Al Amiri hospital, Kuwait | | | | |
| --- | --- | --- | --- | --- |
| Antibiotic | Range (µg/ml) | MIC 50 | MIC90 | No. (% )resistant |
| Ampicillin (AMP) | 0.125 - 256 | 3 | 256 | 11 (35.5) |
| Ceftazidime (CAZ) | 0.094 - 32 | 0.25 | 2 | 2 (6.5) |
| Cefatoxime (CTX) | 0.94 - 32 | 0.125 | 0.25 | 2 (6.5) |
| Ceftriaxone (CRO) | 0.032 - 256 | 0.064 | 0.125 | 2 (6.5) |
| Imipenem (IPM) | 0.032 – 1.0 | 0.19 | 0.25 | 0 (0) |
| Meropenem(MEM) | 0.012 - 0.125 | 0.032 | 0.047 | 0 (0) |
| Piperacillin/Tazobactam (TZP) | 1.00 - 8 | 2 | 6 | 0 (0) |
| Tetracycline (TET) | 2 - 256 | 6 | 256 | 15 (48.4) |
| Gentamicin (GM) | 0.38 - 1 | 0.5 | 0.75 | 1 (3.2) |
| Trimethoprim - Sulfamethoxazole (SXT) | 0.094 -32 | 0.25 | 32 | 4 (12.9) |
| Chloramphenicol (CHL) | 3 - 256 | 6 | 12 | 2 (6.5) |
| Ciprofloxacin (CIP) | 0.016 - 1.5 | 0.032 | 0.5 | 8 (25.8) |
